# Allergen-specific IgE and IgG4 patterns among patients with different allergic diseases

**DOI:** 10.1186/s40413-018-0220-5

**Published:** 2018-12-03

**Authors:** Olga Smoldovskaya, Guzel Feyzkhanova, Sergei Voloshin, Alla Arefieva, Antonina Chubarova, Ludmila Pavlushkina, Tatiana Filatova, Eugenia Antonova, Elena Timofeeva, Veronika Butvilovskaya, Yuri Lysov, Alexander Zasedatelev, Alla Rubina

**Affiliations:** 1 Engelhardt Institute of Molecular Biology, Russian Academy of Sciences, Vavilova str. 32, Moscow, Russian Federation 119991; 2 Filatov Moscow City Pediatric Clinic No. 13, Moscow, Russia

**Keywords:** Allergy diagnostics, IgE, IgG4, Microarrays, Sensitization, Asthma, Dermatitis, Rhinitis

## Abstract

**Background:**

In addition to allergen-specific IgE (sIgE), allergen-specific IgG4 (sIgG4) antibodies are also involved in the immune response resulting from an allergen exposure. The aim of our study was to analyze sIgE and sIgG4 patterns in the most common allergic disorders: bronchial asthma, upper airway disorders and atopic dermatitis.

**Methods:**

In this study a screening analysis of blood serum samples from 673 patients aged from 6 months to 17 years with different allergic entities was performed on microarrays. sIgE and sIgG4 levels to the most common allergens were estimated.

**Results:**

sIgE response to most pollen allergens is more strongly associated with respiratory diseases than with atopic dermatitis, while sIgE responses to cat and dog dander are more strongly associated with bronchial asthma than with atopic dermatitis and upper airway disorders such as rhinosinusitis and allergic rhinitis. A lower prevalence of sIgG4 to pollen allergens in cases of atopic dermatitis is observed compared with that in cases of asthma and upper airway disorders. Analyzing all the allergic disorders, one can see that sIgG4 response to inhalant allergens is strongly associated with sensitization to the corresponding allergen.

**Conclusion:**

Allergen-specific IgE and IgG4 patterns that are relevant to concrete allergic diseases differ by sIgE and sIgG4 prevalences to defined allergens.

**Electronic supplementary material:**

The online version of this article (10.1186/s40413-018-0220-5) contains supplementary material, which is available to authorized users.

## Background

Bronchial asthma, allergic rhinitis and atopic dermatitis are the most common allergic reactions. The pathologies individually and their comorbidities are often diagnosed among many atopic patients in different periods of life [1]. Along with genetics and adverse environmental factors, allergens as triggering factors represent major aspects of the pathogenic pathways for the mentioned entities [2–4].

Currently, allergen-specific IgE is considered the only notable serological marker of type 1 hypersensitivity. A number of studies [5, 6] have focused on sIgE responses among children and adults with different allergic pathologies. However, most of the studies have assessed only one particular pathology or only one particular group of allergens [7, 8].

Allergen-specific IgG, including subclass G4, participates in the development of allergic reactions. The analysis of allergen-specific IgG4 is mainly performed in studies that are related to allergen-specific immunotherapy (ASIT) [9], and a change in the sIgG4 level is one of the indicators of the efficiency of ASIT, during which allergen tolerance occurs [10]. However, in some cases, similar processes of tolerance induction with the involvement of sIgG4, as a part of the whole pool of allergen-specific IgG, occur among subjects not treated with ASIT [11]. The most representative example is the development of tolerance to food allergens while growing up [12]. However, the sIgG4 response among atopic patients of different age, states and diseases was less studied, and IgG4 antibodies are regarded as minor party in hypersensitivity reaction manifestation because of low serum concentrations.

The aim of our study was to analyze the sIgE and sIgG4 responses to allergens that are most relevant to the chosen allergic pathologies. We determined sIgE and sIgG4 levels in blood serum samples from 673 patients diagnosed with bronchial asthma, upper airway disorders or atopic dermatitis using microarrays with immobilized protein extracts of 31 allergens. Profiles of the groups with different allergic pathologies were defined, and the correlation between sIgE and sIgG4 occurrence was evaluated.

## Methods

### Participants and samples

Patients from the Filatov Moscow City Pediatric Clinic No. 13 aged from 6 months to 17 years were enrolled in the study (Table 1). The recruitment was carried out from September, 2016, to September, 2017. Participants chosen for the study had three different diagnosis according to clinical history and medical examination: 1) atopic patients without dermatitis symptoms with lower airway disease or bronchial asthma (145 patients), 2) atopic patients with upper airway disorders (rhinosinusitis and allergic rhinitis) without asthma and dermatitis symptoms (194 patients), 3) participants with atopic dermatitis without symptoms of airway disorders (334 patients) (Table 1).

**Table 1:** Characterization of the participants of the current study

Allergic disorders	Male			Female		
	0–6 years	7–12 years	13–17 years	0–6 years	7–12 years	13–17 years
Asthma	28	37	36	9	21	14
Upper airway disorders	26	44	36	28	30	30
Dermatitis	104	38	11	134	35	12

The patients included in groups with airway disorders (bronchial asthma and upper airway disorders) had not demonstrated symptoms of allergic skin reaction for a period of 1 year before the study. The patients in the group with atopic dermatitis had not demonstrated symptoms of allergic airway diseases for a period of 1 year before the study. The children with well controlled and/or well-medicated asthma were not included in the group with atopic dermatitis.

Surplus blood serum samples that remained after routine diagnostic procedures were analyzed. Additional blood draws were not performed. The study was approved by the local ethics committee of the Filatov Moscow City Pediatric Clinic No. 13.

### Microarray design and manufacturing

**Fig. 1:**
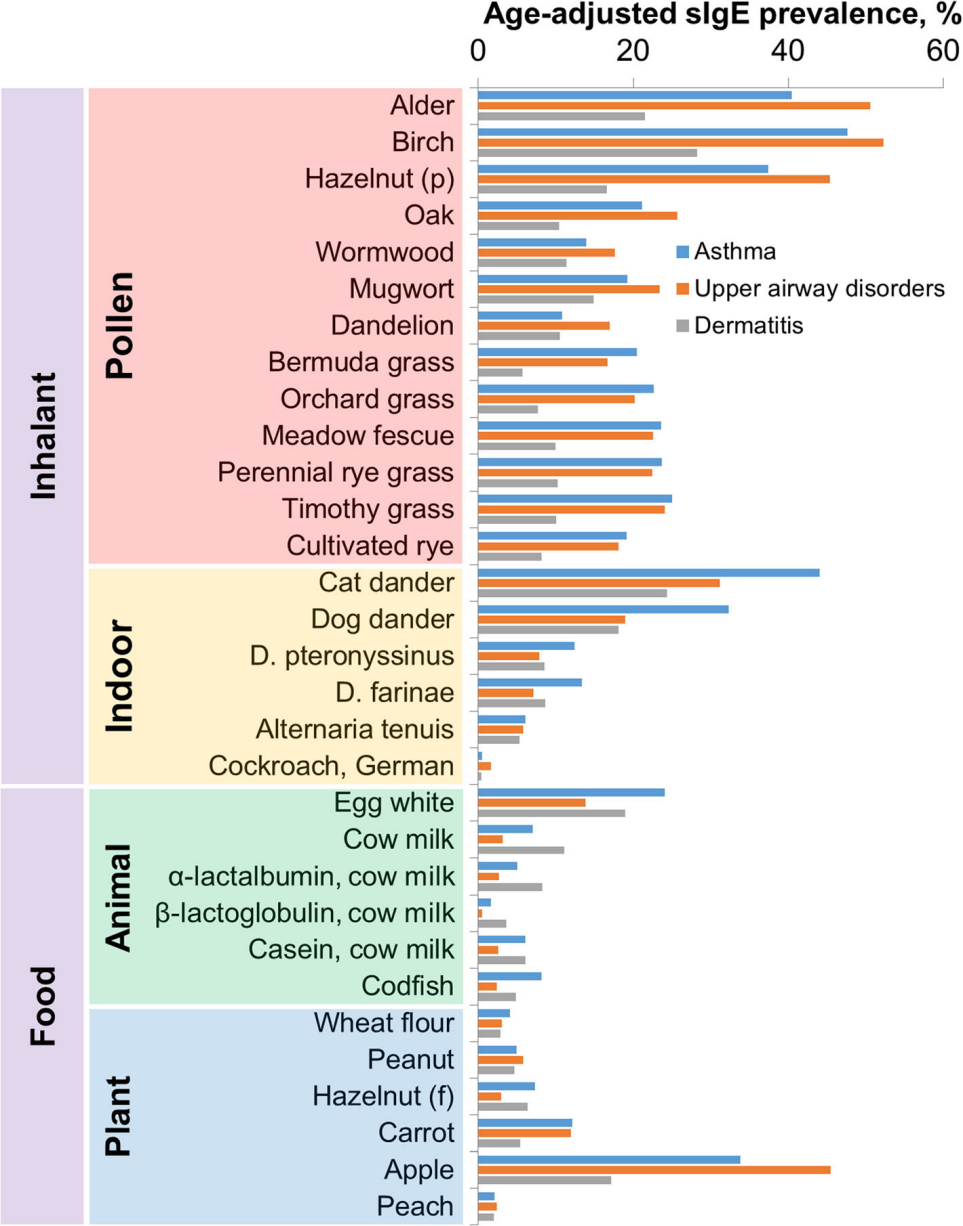
Age-adjusted sIgE prevalences (the rate of patients who exhibit sIgE above 0.35 IU/ml) among the patients diagnosed with bronchial asthma, upper airway disorders (such as rhinosinusitis and rhinitis) or atopic dermatitis

Microarray design and manufacturing technologies were described in full in the previous works [13, 14]. Briefly, the microarray is a matrix of semispherical hydrogel elements (0.1 nl in volume) that contain one of the immobilized allergens: 28 allergen extracts and 3 individual isolated proteins (components of cow milk) (for the list of allergens see Fig. 1). The allergens for immobilization were purchased from GREER (Lenoir, NC, USA) and were diluted to working concentrations (from 1 to 5 mg/ml depending on the allergen) according to the manufacturer’s recommendations. The list of allergens includes most common allergens in Central Russia [14]; this list largerly overlaps with the widespread allergens in Central and Northern Europe.

Concentrations of the allergens were chosen in such a way that the results obtained from the microarrays most accurately coincided with the results obtained by the reference methods (Specific IgE REAST (ALLERG-O-LIQ) and Specific IgG4 ELISA (Dr. Fooke Laboratorien GmbH, Germany)) [14]. The sIgE levels were determined in the range of 0.35–100 IU/ml, and the sIgG4 levels were determined in the range of 100–2500 ng/ml.

### Analysis of allergen-specific IgE and IgG4

The analysis of sIgE and sIgG4 on the microarrays includes 4 stages: 1) incubation of the microarray with the serum sample; 2) washing with a washing buffer containing detergent; 3) incubation with the developing antibodies, a mixture of anti-human IgE and anti-human IgG4 antibodies conjugated with Cy5 and Cy3, respectively; 4) washing with a washing buffer containing detergent [14].

After the analysis, fluorescent signals from the gel pads with immobilized allergens are detected using a microarray analyzer with laser illumination and a device for speckle suppression [15]. Fluorescent signals are calculated by referencing piecewise linear calibration curves constructed on the basis of the signals from the gel pads with immobilized IgE and IgG4 to obtain the sIgE and sIgG4 concentrations in IU/ml and ng/ml, respectively.

Patients were considered sensitized to the allergen if the sIgE level for the allergen exceeded the minimum cutoff of 0.35 IU/ml. Nonsensitized, monosensitized and polysensitized patients were enrolled in the study.

### Data evaluation

sIgE prevalence was determined as the ratio of patients who exhibit sIgE above the cutoff of 0.35 IU/ml to the total number of patients in the group of interest. sIgG4 prevalence was determined as the ratio of patients who exhibit sIgG4 above the value of 100 ng/ml to the total number of patients in the group of interest.

sIgE prevalence and sIgG4 prevalence were adjusted by age with the average population as a standard [16] using the Epitools package [17] in R [18]. Relationships between allergic sensitization and diseases were analyzed using a logistic regression model. To compare sIgE and sIgG4 prevalences among patients with different diseases, adjusted odds ratios (aORs) and corresponding 95% confidence intervals (CIs) were calculated via multinomial logistic regression analysis with the age and gender as covariates using IBM SPSS Statistics 23.0.0.0.

The statistical significance of the differences in sIgG4 response for sensitized and non-sensitized patients was estimated according to the results of the Fisher’s exact test calculated by MedCalc. The differences were considered statistically significant for pairwise comparisons with *p* < 0.05.

The diagrams were plotted using Microsoft Excel 2010.

## Results

In the current study, sIgE and sIgG4 levels of 673 blood serum samples from patients aged from 6 months to 17 years with allergic reactions (bronchial asthma – 145 samples, upper airway disorders – 194 samples, and atopic dermatitis – 334 samples) were obtained.

Results of the determination of allergen-specific IgE and IgG4 as well as sIgE and sIgG4 prevalence for different allergens in different age groups are presented in Additional file 1. Sensitization rates to each selected allergen, adjusted by age for different patient groups, are presented in Fig. 1. The most significant differences between sIgE prevalences in different diseases are observed for inhalant allergens. For these allergens, ORs adjusted by age and gender are shown in Table 2.

**Table 2:** Adjusted ORs (aORs) for sIgE and sIgG4 prevalences among patients diagnosed with bronchial asthma, upper airway disorders (such as rhinosinusitis and rhinitis) or atopic dermatitis for inhalant allergens

Allergens	Asthma versus Upper Airway Disorders		Asthma versus Dermatitis		Upper Airway Disorders versus Dermatitis	
	aOR	95% CI	aOR	95% CI	aOR	95% CI
sIgE to inhalant allergens						
Pollen allergens						
Alder	0.809	(0.521;1.257)	2.980	(1.863;4.766)	3.682	(2.384;5.684)
Birch	1.015	(0.653;1.578)	2.705	(1.725;4.242)	2.664	(1.76;4.032)
Hazelnut (p)	0.889	(0.571;1.385)	3.700	(2.251;6.081)	4.160	(2.617;6.611)
Oak	0.921	(0.568;1.494)	3.086	(1.714;5.555)	3.349	(1.928;5.815)
Wormwood	0.849	(0.487;1.478)	1.253	(0.669;2.349)	1.477	(0.83;2.629)
Mugwort	0.856	(0.518;1.415)	1.395	(0.8;2.434)	1.629	(0.979;2.712)
Dandelion	0.668	(0.37;1.208)	1.031	(0.52;2.041)	1.542	(0.844;2.819)
Bermuda grass	1.460	(0.873;2.44)	5.192	(2.569;10.491)	3.556	(1.792;7.055)
Orchard grass	1.265	(0.772;2.074)	3.740	(1.999;6.997)	2.956	(1.622;5.385)
Meadow fescue	1.146	(0.705;1.865)	3.179	(1.754;5.761)	2.773	(1.577;4.875)
Perennial rye grass	1.139	(0.701;1.852)	3.075	(1.706;5.543)	2.698	(1.543;4.718)
Timothy grass	1.123	(0.697;1.81)	3.405	(1.904;6.091)	3.032	(1.747;5.262)
Cultivated rye	1.145	(0.685;1.913)	2.601	(1.389;4.868)	2.272	(1.249;4.133)
Indoor allergens						
Cat dander	1.808	(1.154;2.833)	2.344	(1.461;3.761)	1.411	(0.897;2.221)
Dog dander	2.225	(1.365;3.628)	2.401	(1.451;3.975)	1.079	(0.646;1.803)
*D. pteronyssinus*	1.390	(0.735;2.629)	1.478	(0.72;3.032)	1.063	(0.522;2.165)
*D. farinae*	1.802	(0.947;3.431)	1.760	(0.873;3.549)	0.976	(0.474;2.012)
*Alternaria tenuis*	1.212	(0.552;2.665)	1.243	(0.525;2.942)	1.025	(0.452;2.326)
Cockroach, German	0.218	(0.025;1.909)	0.886	(0.048;16.465)	4.065	(0.406;40.654)
sIgG4 to inhalant allergens						
Pollen allergens						
Alder	1.102	(0.679;1.787)	3.311	(1.845;5.943)	3.006	(1.728;5.229)
Birch	0.864	(0.557;1.338)	2.379	(1.505;3.76)	2.754	(1.807;4.199)
Hazelnut (p)	0.941	(0.547;1.618)	2.643	(1.358;5.146)	2.810	(1.505;5.248)
Oak	0.925	(0.381;2.246)	4.063	(1.204;13.71)	4.391	(1.412;13.652)
Wormwood	0.942	(0.432;2.058)	2.963	(1.048;8.383)	3.144	(1.193;8.288)
Mugwort	1.147	(0.624;2.109)	1.938	(0.97;3.873)	1.690	(0.875;3.263)
Dandelion	0.339	(0.132;0.87)	0.811	(0.275;2.388)	2.390	(1.057;5.407)
Bermuda grass	0.446	(0.119;1.67)	1.371	(0.301;6.245)	3.076	(0.972;9.735)
Orchard grass	1.140	(0.337;3.854)	2.593	(0.598;11.248)	2.274	(0.566;9.139)
Meadow fescue	1.189	(0.473;2.992)	5.462	(1.32;22.598)	4.593	(1.168;18.064)
Perennial rye grass	1.745	(0.645;4.721)	4.491	(1.215;16.598)	2.573	(0.693;9.556)
Timothy grass	0.926	(0.395;2.171)	2.673	(0.855;8.358)	2.888	(0.998;8.358)
Cultivated rye	0.395	(0.08;1.952)	2.725	(0.345;21.526)	6.896	(1.303;36.492)
Indoor allergens						
Cat dander	1.496	(0.913;2.453)	1.671	(0.992;2.814)	1.117	(0.673;1.852)
Dog dander	1.067	(0.607;1.876)	1.813	(0.975;3.372)	1.699	(0.948;3.045)
*D. pteronyssinus*	0.984	(0.469;2.063)	1.661	(0.75;3.682)	1.688	(0.804;3.544)
*D. farinae*	0.681	(0.314;1.479)	1.333	(0.568;3.128)	1.957	(0.932;4.109)
*Alternaria tenuis*	0.387	(0.08;1.876)	0.687	(0.127;3.705)	1.775	(0.568;5.553)
Cockroach, German	0.264	(0.03;2.311)	1.152	(0.084;15.738)	4.362	(0.662;28.717)

aORs were calculated using logistic regression analysis with the age and gender as covariates

Patients with airway disorders (bronchial asthma and upper airway disorders) are mostly sensitized to pollen allergens. Notably, the adjusted sIgE prevalence for most pollen allergens distinguishes less than 10% for the patients with asthma and with upper airway disorders (Fig. 1). Sensitization to the majority of pollen allergens is more associated with airway diseases than with atopic dermatitis (i.e. aOR is significantly higher than 1, see Table 2).

Indoor allergens also have a significant impact, especially the animal epithelium allergens of cat dander and dog dander. sIgE sensitization to these allergens is more common for patients with bronchial asthma than for patients affected by only upper airway disorders or atopic dermatitis (Fig. 1). For these cases, as shown in Table 2, when comparing bronchial asthma and upper airway disorders for cat dander aOR = 1.808, 95% CI (1.154;2.833), for dog dander aOR = 2.225, 95% CI (1.365;3.628); when comparing bronchial asthma and atopic dermatitis for cat dander aOR = 2.344, 95% CI (1.461;3.761), for dog dander aOR = 2.401, 95% CI (1.451;3.975).

The adjusted prevalence of sensitization to food allergens of animal origin is higher in asthma and atopic dermatitis patients than in patients with upper airway disorders. However, this difference is most commonly not statistically significant (i.e. 95% CI for aORs contains 1, Table 3). Sensitization to a number of plant food allergens (hazelnut, carrot, peach and apple) is observed mostly for patients sensitized to tree pollens. It is strongly influenced by pollen sensitization, because mentioned allergens contain major components homologous to major tree pollen proteins from Bet v1-like family [19, 20].

**Table 3:** Adjusted ORs (aORs) for sIgE and sIgG4 prevalences among patients diagnosed with bronchial asthma, upper airway disorders (such as rhinosinusitis and rhinitis) or atopic dermatitis for food allergens

Allergens	Asthma versus Upper Airway Disorders		Asthma versus Dermatitis		Upper Airway Disorders versus Dermatitis	
	aOR	95% CI	aOR	95% CI	aOR	95% CI
sIgE to food allergens						
Animal food allergens						
Egg white	1.782	(0.977;3.25)	1.693	(0.976;2.939)	0.950	(0.55;1.64)
Cow milk	1.526	(0.518;4.499)	0.645	(0.253;1.646)	0.423	(0.168;1.066)
α-lactalbumin, cow milk	1.057	(0.289;3.866)	0.527	(0.165;1.686)	0.499	(0.181;1.374)
β-lactoglobulin, cow milk	3.264	(0.286;37.209)	0.852	(0.183;3.96)	0.261	(0.033;2.049)
Casein, cow milk	1.227	(0.363;4.149)	0.875	(0.293;2.619)	0.714	(0.26;1.962)
Codfish	3.560	(1.215;10.432)	2.646	(1.039;6.739)	0.743	(0.237;2.329)
Plant food allergens						
Wheat flour	1.442	(0.464;4.483)	1.935	(0.64;5.85)	1.342	(0.472;3.811)
Peanut	1.455	(0.528;4.01)	1.290	(0.494;3.367)	0.887	(0.345;2.278)
Hazelnut (f)	2.874	(1.059;7.798)	1.626	(0.687;3.847)	0.566	(0.202;1.587)
Carrot	1.075	(0.587;1.969)	3.089	(1.449;6.584)	2.873	(1.388;5.946)
Apple	0.789	(0.506;1.23)	3.164	(1.927;5.195)	4.011	(2.535;6.346)
Peach	0.772	(0.179;3.327)	1.863	(0.392;8.866)	2.413	(0.609;9.561)
sIgG4 to inhalant allergens						
Animal food allergens						
Egg white	0.297	(0.091;0.973)	2.305	(1.06;5.014)	7.762	(2.666;22.6)
Cow milk	1.001	(0.557;1.803)	1.493	(0.841;2.653)	1.490	(0.878;2.531)
α-lactalbumin, cow milk	0.865	(0.537;1.395)	1.262	(0.789;2.02)	1.458	(0.94;2.264)
β-lactoglobulin, cow milk	1.105	(0.688;1.776)	1.765	(1.106;2.818)	1.597	(1.048;2.437)
Casein, cow milk	1.213	(0.76;1.939)	2.037	(1.273;3.262)	1.678	(1.1;2.56)
Codfish	1.288	(0.623;2.663)	3.841	(1.613;9.151)	2.981	(1.285;6.92)
Plant food allergens						
Wheat flour	1.164	(0.75;1.809)	1.960	(1.262;3.044)	1.683	(1.128;2.512)
Peanut	0.974	(0.622;1.524)	1.823	(1.134;2.931)	1.871	(1.206;2.905)
Hazelnut (f)	0.919	(0.594;1.425)	1.971	(1.257;3.093)	2.144	(1.418;3.243)
Carrot	1.261	(0.68;2.338)	2.137	(1.08;4.232)	1.694	(0.878;3.269)
Apple	1.090	(0.667;1.781)	2.004	(1.171;3.43)	1.838	(1.112;3.041)
Peach	1.348	(0.774;2.351)	2.593	(1.428;4.711)	1.923	(1.088;3.4)

aORs were calculated using logistic regression analysis with the age and gender as covariates

**Fig. 2:**
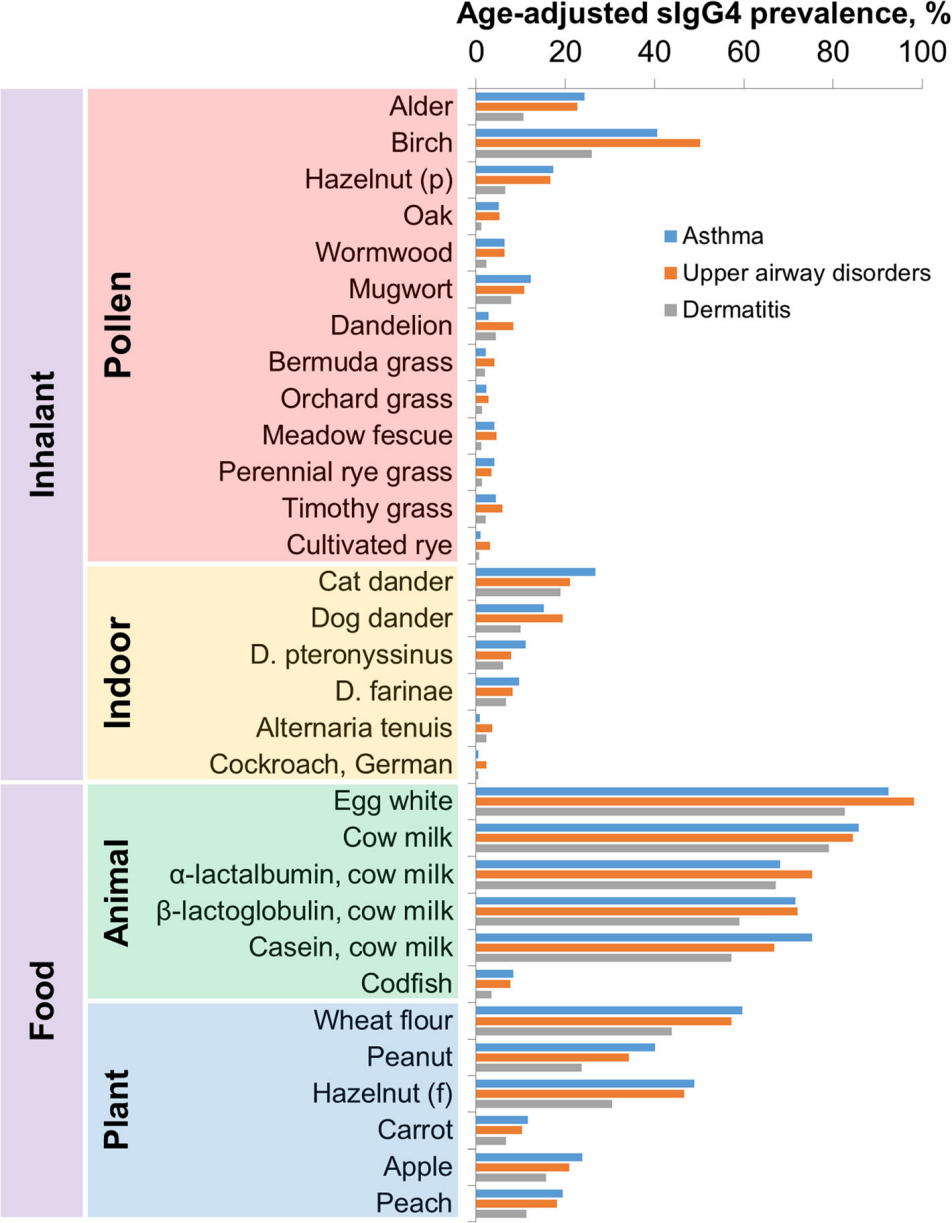
Age-adjusted sIgG4 prevalences (the rate of patient who exhibit sIgG4 above 100 ng/ml) among the patients diagnosed with bronchial asthma, upper airway disorders (such as rhinosinusitis and rhinitis) or atopic dermatitis

sIgG4 responses to food allergens are observed in patients with all allergic diseases (Fig. 2). Among inhalant allergens, the highest adjusted sIgG4 prevalence is observed for the allergens most responsible for sensitization, i.e. detectable sIgE production (birch pollen, alder pollen, cat dander, and dog dander).

**Fig. 3:**
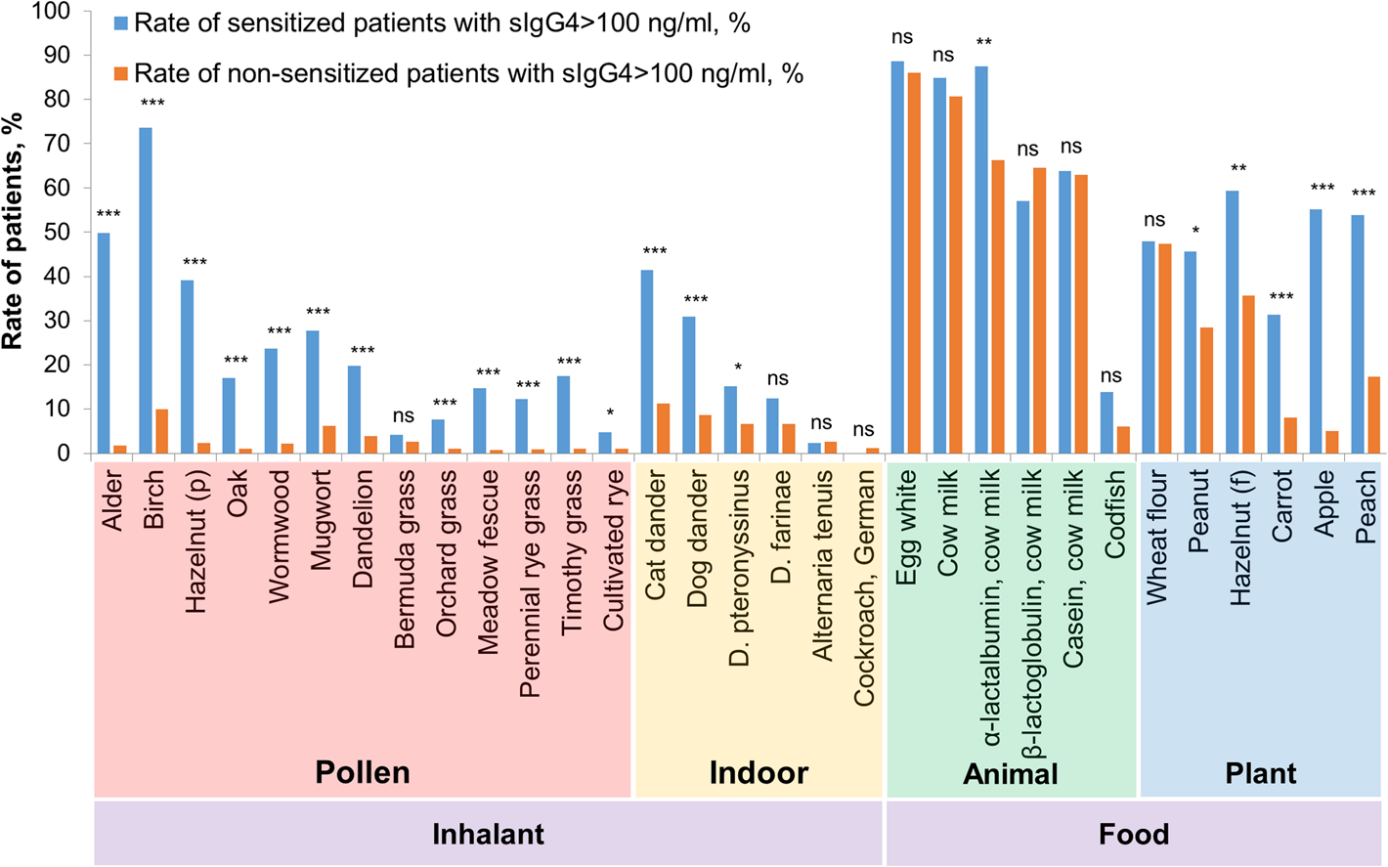
The rate of patients with sIgG4 > 100 ng/ml among sensitized and non-sensitized to each allergen groups of patients. Comparison of proportions by the Fisher exact test is noted by *p*-values: * – *p* < 0.05; ** – *p* < 0.01, *** – *p* < 0.001

Figure 3 indicates that detectable sIgG4 levels to inhalant allergens and some plant food allergens are common in sensitized patients with detectable sIgE levels, whereas no reliable difference is observed in rates of patients with sIgG4 to food allergens of animal origin between sensitized and non-sensitized groups.

Significant differences in adjusted sIgG4 prevalence are observed between the groups with atopic dermatitis and with airway disorders for a variety of inhalant (mostly pollen) and food allergens (Tables 2 and 3).

## Discussion

This study is aimed at the sIgE and sIgG4 responses among atopic patients from 6 months to 17 years of age with allergic diseases: bronchial asthma, upper airway disorders or atopic dermatitis. The age range was chosen as 0 to 17 years because it is known that there are noticeable changes in immunoglobulin patterns among allergic patients during this period of life [21, 22]. After the age of 18–20 years the sIgE profiles do not change significantly [23].

Although genetic predispositions to asthma and upper airway disorders are associated with different genetic polymorphisms, both diseases have common profiles of inflammatory mediators and cell-mediated responses involving eosinophils in the allergic inflammation [24]. Subsequently, the corresponding similarity in the physiological processes affects the prevalence of sIgE to the allergens involved.

For all explored allergens, with few exceptions, namely, cat and dog dander, considerable differences between sIgE profiles for patients with asthma and upper airway disorders are not observed (Fig. 1). Daniel J. Stoltz et al. [25] reported that increased asthma risk at the age of 6 years is more strongly associated with sensitization to dog and cat dander at 1 and 3 years than with the absence of sensitization at younger age. Similar interrelation is not observed for allergic rhinitis; that is why the authors suggested that sensitization to perennial allergens is more closely linked to asthma development. Our investigations are in agreement: sIgE sensitization to cat or dog dander are more strongly associated with asthma then with upper airway disorders (for cat dander – aOR = 1.808, 95% CI (1.154;2.833); for dog dander – aOR = 2.225, 95% CI (1.365;3.628)).

Our data on sIgE sensitization partly coincides with the results observed among adult population (22–86 years old) in the study [26]: there were no significant differences in sIgE prevalences between patients with asthma and rhinitis not only for pollen allergens but for cat and dog dander too.

Unlike upper airway disorders, atopic dermatitis and bronchial asthma have a strong connection with food allergens [27, 28], which agrees with our results: sIgE prevalences for food allergens are higher for these pathologies, though the difference is not statistically significant, probably because of the insufficient sampling size.

As the study has not included healthy controls, we have compared sIgE prevalence in our study with the sIgE prevalence in random population from Russian Karelia, that was analyzed in the study of Ruokolainen L et al. [29] (Additional file 2 in Supplementary materials). From the Additional file 2 one can see that sensitization rate for analyzed inhalant allergens in a random population is lower than normalized sensitization rate in the groups with atopic patients from our study. As the random population from the study of Ruokolainen L et al. included some atopic patients (5,1% with atopic eczema, 1,2% with asthma) we assume that the sensitization rate in the group containing only healthy donors would be even lower, so the mentioned tendency would be correct for the control group of healthy donors too.

In contrast to IgE, the functions of IgG antibodies of different subclasses in the allergic pathologic process are still being discussed. The food-specific IgG response of different subclasses is mostly considered as part of the normal reaction to natural exposure to food products [11, 30]. Food allergens are the most common antigens that lead to the production of sIgG, and its subclass sIgG4 accordingly [31]. The data regarding sIgG4 obtained in our study confirm these findings (Fig. 2). Previously, we have studied the changes in sIgG4 prevalence among pediatric patients depending on the age and our results showed that the prevalence of sIgG4 to food antigens increases throughout the age [22]. According to a number of studies [12, 32], this increase might be the hallmark of tolerance induction.

Notably, IgG4 represents a small portion of total IgG. Thus, the evolution of IgG in the young population is not associated with the evolution of IgG4 but with the IgG1 subclass, which is the main fraction of IgG [33]. Xinyuan Huang et al. [31] demonstrated that the production of the total IgG specific to inhalant allergens correlates with sensitization to the antigen in question. Schwarz A et al. [30] showed that sIgG to inhalant allergens as well as sIgG4 subclass to a variety of food allergens are observed mostly among sensitized patients at the age of 2 years. In [30] sIgG4 to inhalant allergens was not observed due to the extremely low sIgG4 prevalence to these antigens (< 5%) at the studied age.

The median age of the participants in our study is higher than that in [30], which corresponds to the increased sIgG4 prevalence in our cohort; it explains noticeable sIgG4 prevalence to inhalant allergens observed in the present experiments. Our study is focused only on sIgG4, however, the present analysis of sIgG4 alone is also of interest because subjects with the prevailing sIgG4 response are usually present along with the subjects with the prevailing sIgG1 response [34].

In the present study, a significant difference is observed in the rates of patients with sIgG4 response between the groups of sensitized and non-sensitized patients for most inhalant allergens assessed (Fig. 3). In contrast, significant differences for food allergens that are analogous to the food components in the study [30] (β-lactoglobulin – Bos d5, casein – Bos d8) were not affirmed. This finding can be explained by the evolution of sIgG4 as subjects age.

The observed interrelation of sIgG4 and sIgE responses for inhalant allergens combined with the differences in sIgE profiles between patients with airway disorders and those with atopic dermatitis leads to significant distinctions in the sIgG4 profiles for pollen allergens, for which significant differences in adjusted sIgE prevalence were observed while comparing different allergic diseases (Table 2).

Due to the positive correlation between the presence of sIgE and significant sIgG4 to inhalant allergens in the serum samples, we assume that lower sensitization rates to inhalant allergens in healthy donors in comparison with atopic patients lead to the lower rates of sIgG4 response to inhalant allergens in the group of healthy population. Similar observation was made by Dubakienė et al. [35] for one of the inhalant allergens, *D. pteronyssinus*.

Overall, this analysis of sIgE response to inhalant allergens among atopic patients shows that sIgE prevalence to pollen allergens is significantly reduced in patients with atopic dermatitis compared with those with asthma and upper airway disorders. In addition, cat and dog sensitization are more strongly associated with bronchial asthma than with atopic dermatitis and upper airway disorders. The increased sIgG4 response to pollen allergens among patients with respiratory allergic diseases can be considered a consequence of the fact that sIgG4 response is most common among patients with detectable sIgE and of the features of the sIgE profiles for these pathologies, as illustrated by experimental data analysis in our study.

## Conclusions

The obtained data demonstrate that different allergic disease entities are characterized by individual features of allergen-specific IgE and IgG4 production, which leads to specific manifestations of allergic inflammation. A more comprehensive study of patients’ sIgE and sIgG4 profiles with respect to the clinical performance and functions of allergen-specific antibodies would allow more effective diagnosis and treatment of atopic patients.

## Additional files

Additional file 1: Results of the determination of allergen-specific IgE and IgG4 on the microarrays. The data are shown in qualitative format: 0 - antibody concentration do not reach the cutoff of 0.35 IU/ml for sIgE or the value of 100 ng/ml for sIgG4. 1 - antibody concentration reach the cutoff of 0.35 IU/ml for sIgE or the value of 100 ng/ml for sIgG4. Spreadsheet “Age groups”: The rate of patients with sIgE > 0.35 IU/ml or sIgG4 > 100 ng/ml, %. The data were normalized in each cell to the number of patients with the indicated diseases in the indicated age group. (XLSX 168 kb)

Additional file 2: Comparison of age-adjusted sIgE prevalences among the patients diagnosed with bronchial asthma, upper airway disorders (such as rhinosinusitis and rhinitis) or atopic dermatitis involved in the study with the sIgE prevalences of the random population from Russian Karelia (Ruokolainen L et al., Clin Exp Allergy. 2017). (TIF 96 kb)

## Abbreviations

aOR: Adjusted odds ratio
ASIT: Allergen-specific immunotherapy
CI: Confidence interval
sIgE: Specific immunoglobulin E
sIgG4: Specific immunoglobulin G4
