# Supplementary figures and images for: Allergen-specific IgE and IgG4 patterns among patients with different allergic diseases
Source: World Allergy Organ J. 2018 Dec 3;11(1):35. doi: 10.1186/s40413-018-0220-5 (PMC6276220; doi:10.1186/s40413-018-0220-5)

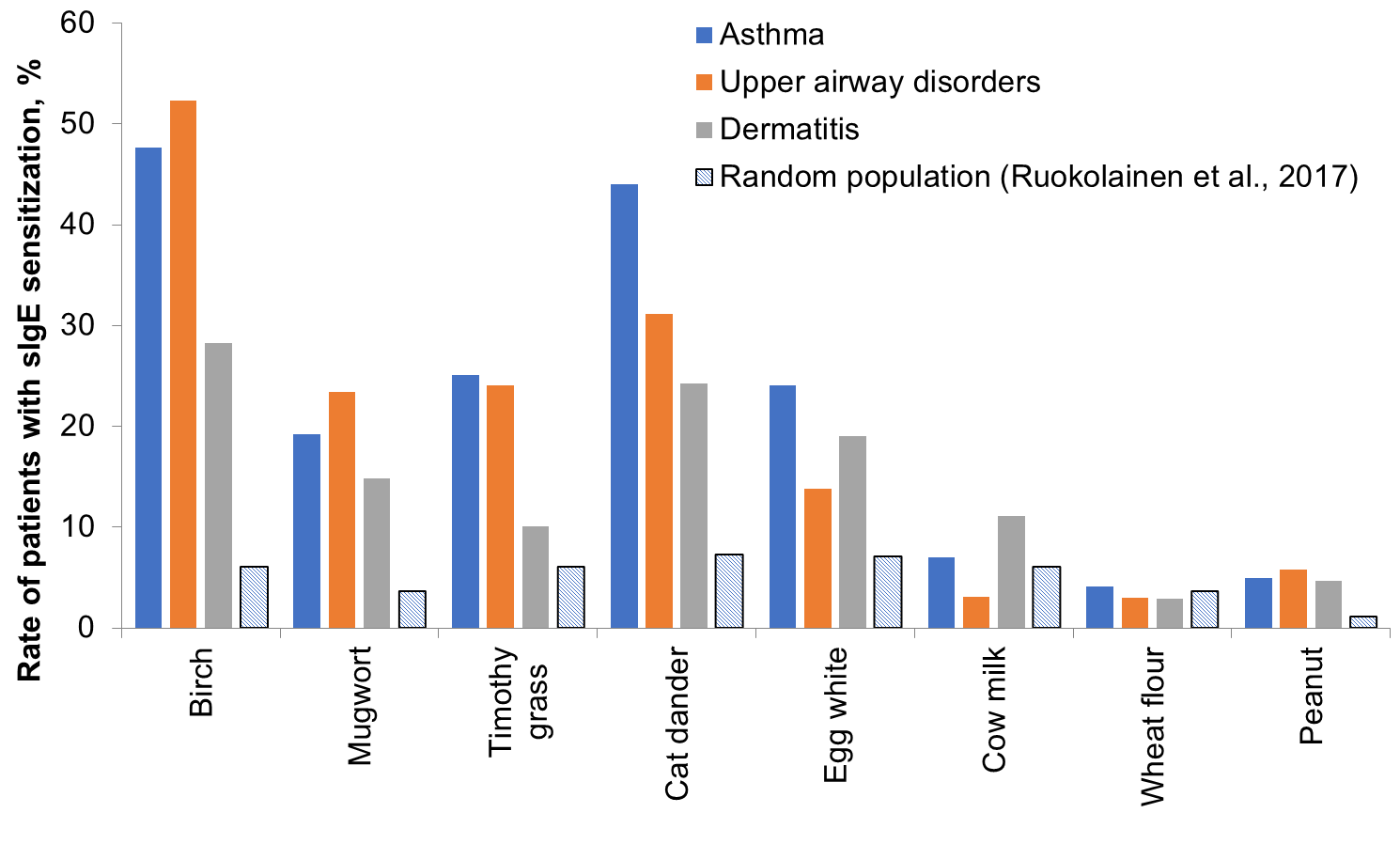

Supplement: Supplementary file 2 — Comparison of age-adjusted sIgE prevalences among the patients diagnosed with bronchial asthma, upper airway disorders (such as rhinosinusitis and rhinitis) or atopic dermatitis involved in the study with the sIgE prevalences of the random population from Russian Karelia (Ruokolainen L et al., Clin Exp Allergy. 2017). (TIF 96 kb) [file 40413_2018_220_MOESM2_ESM.tif]
